# Unveiling the Atomic and Electronic Structure of Stacked-Cup Carbon Nanofibers

**DOI:** 10.1186/s11671-021-03595-y

**Published:** 2021-10-11

**Authors:** D. W. Boukhvalov, I. S. Zhidkov, A. Kiryakov, J. L. Menéndez, L. Fernández-García, A. I. Kukharenko, S. O. Cholakh, A. F. Zatsepin, E. Z. Kurmaev

**Affiliations:** 1 College of Science, Institute of Materials Physics and Chemistry, Nanjing Forestry University, Nanjing, 210037 People’s Republic of China; 2 Institute of Physics and Technology, Ural Federal University, Yekaterinburg, Russia 620002; 3 Centro de Investigación en Nanomateriales Y Nanotecnología, Consejo Superior de Investigaciones Científicas (CSIC)—Universidad de Oviedo (UO)—Principado de Asturias, Avenida de la Vega 4-6, El Entrego, 33940 San Martin del Rey Aurelio, Asturias Spain; 4 M.N. Mikheev Institute of Metal Physics, Russian Academy of Sciences, Ural Branch, Yekaterinburg, Russia 620108

**Keywords:** Carbon nanofibers, XPS, DFT, Nanocarbons, Quantum dots, Photoluminescence

## Abstract

We report results of comprehensive experimental exploration (X-ray photoemission, Raman and optical spectroscopy) of carbon nanofibers (CNFs) in combination with first-principles modeling. Core-level spectra demonstrate prevalence of *sp*2 hybridization of carbon atoms in CNF with a trace amount of carbon–oxygen bonds. The density functional theory (DFT)-based calculations demonstrated no visible difference between mono- and bilayers because *σ*-orbitals are related to in-plane covalent bonds. The influence of the distortions on *π*-peak is found to be significant only for bilayers as a result of *π*–*π* interlayer bonds formation. These results are supported by both experimental Raman and XPS valence band spectra. The combination of optical measurements with a theoretical modeling indicates the formation of optically active graphene quantum dots (GQDs) in the CNF matrix, with a radiative relaxation of the excited π* state. The calculated electronic structure of these GQDs is in quantitative agreement with the measured optical transitions and provides an explanation of the absence of visible contribution from these GQDs to the measured valence bands spectra.

## Introduction

Carbon nanofibers (CNFs) are nanofilaments (from 3 to 100 nm in diameter) organized by stacked graphene layers with a certain orientation with respect to the fiber axis. CNFs have a great potential as promising materials in photonic and electronic devices, optical sensors, electrode materials for batteries and supercapacitors, new reinforcing composites and other functional materials due to high ratio of surface area to volume, nanoscale diameter of carbon particles and superior mechanical, electrical and chemical properties [1]. Due to their high electrical and thermal conductivities as well as their structural properties and surface state, which facilitates functionalization and other surface modification techniques to incorporate the nanofibers to host polymers, CNFs have found applications as secondary/reinforcing phases in different matrices, ranging from ceramics [2, 3], metals [4] and polymers [5–7] to textiles [8]. As a conductive filler, CNFs are found to be more effective than traditional carbon black [9], which results in nanofilled composite materials with high electrical conductivity at lower filler concentrations [10]. At room temperature, the intrinsic resistivity of highly graphitic vapor-grown carbon fibers is approximately 5 × 10^−5^ Ω cm [11], which is comparable to the resistivity of graphite. Recent theoretical modelings propose two opposite models of CNF: as a flat graphene sheet [12, 13] or disordered sponge like carbon foam. [14, 15].

The surface studies are very important for materials such as nanocarbon because their properties tend to dominate at the nanoscale due to the drastically increased the surface-to-volume ratio. The combination of X-ray photoemission spectroscopy (XPS) and density functional theory (DFT)-based modeling is a powerful tool for decryption of atomic structure of nanosized carbons [16]. Taking into account the promising photonic properties of nanostructured carbons [17–19] and the possible formation of graphene quantum dots (GQDs) [20–25] by transformation of the edges graphene layers [23, 24], the combination of additional optical measurements and theoretical modeling is essential for the comprehensive description of the structure and properties of any carbon materials. In this work, we report results of the combination of Raman, XPS, optical measurements of CNF with theoretical modeling of the possible atomic structure of considered materials.

## Methods

Carbon nanofibers from Grupo Antolín Ingeniería (GANF) were produced on an industrial reactor using a Ni catalyst. Ni was solved and introduced continuously into the reactor. A sulfur compound was added to the liquid solution for the production of GANF. Natural gas was used as carbon feedstock with H_2_ as carrier gas at temperatures above 1400 K. Both reactors were externally heated through electrical resistance [26]. The process was optimized to produce stacked-cup CNFs, commercially called GANF. GANF produced this way, with an average fiber diameter of 50 nm and fiber length up to 30 µm, were ball-milled in propanol for 1 h. The dried powder was uniaxially pressed at 30 MPa and compacted at a heating rate of 50 °C min^−1^ in a spark plasma sintering device, model FCT-HP D25/1, under an applied pressure of 80 MPa and in vacuum (10^−1^ mbar). The final compaction temperature was 860 °C and the holding time 1 min.

Raman spectroscopy was performed on a Renishaw 2000 Confocal Raman Microprobe (Renishaw Instruments, England) using a 514.5-nm argon ion laser. The transmission electron microscope (TEM) photographs were obtained with a TEM (JEOL, 2000 FX), and the bulk CNFs sample was previously cut on a Reichert Ultracut E ultramicrotome.

X-ray photoelectron spectra (XPS) were measured using a PHI 5000 Versa Probe XPS spectrometer (ULVAC Physical Electronics, USA) based on a classical X-ray optic scheme with a hemispherical quartz monochromator and an energy analyzer working in the range of binding energies from 0 to 1500 eV. Electrostatic focusing and magnetic screening were used to achieve an energy resolution of Δ*E* ≤ 0.5 eV for the Al K_*α*_ radiation (1486.6 eV). An ion pump was used to maintain the analytical chamber at 10^−7^ Pa, and dual channel neutralization was used to compensate local surface charge generated during the measurements. The XPS spectra were recorded using Al K_*α*_ X-ray emission—spot size was 200 µm, the X-ray power delivered at the sample was less than 50 W, and typical signal-to-noise ratios were greater than 10,000:3.

Optical reflectance spectroscopy was measured on a Lambda 35 spectrophotometer (PerkinElmer) using an integrating sphere. USRS-99-010 was applied as an external standard. A deuterium lamp was used as a source of UV radiation. The spectra were recorded at room temperature.

The photoluminescence spectra were recorded on a Horiba Fluorolog 3 (Jobin Yvon) spectrofluorimeter equipped with a 450 W Xenon lamp and typical signal-to-noise ratios were greater than 20,000:1. A Horiba Synapse CCD camera was installed as a registration detector. The sample was fixed in an adequate holder. The spectra were recorded at room temperature.

For the modeling of the atomic and electronic structure of CNF, the density functional theory (DFT) implemented in the pseudopotential code SIESTA was used, [27] as in our previous studies of similar graphene-based systems [16, 28, 29]. All calculations were performed using the generalized gradient approximation (GGA-PBE) with spin-polarization [30] and implementation of the correction of van der Waals forces [31]. During the optimization, the ion cores were described by norm-conserving non-relativistic pseudo-potentials [32] with cut-off radii 1.14 and the wave functions were expanded with localized orbitals and a double-ζ plus polarization basis set for other species. The atomic positions were fully optimized, and optimization of the force and total energy was performed with an accuracy of 0.04 eV/Å and 1 meV, respectively. All calculations were carried out with an energy mesh cut-off of 300 Ry and a *k*-point mesh of 6 × 6 × 2 and 9 × 9 × 4 in the Monkhorst–Pack scheme [33] for monolayers and bilayers, respectively.

## Results and Discussion

**Fig. 1:**
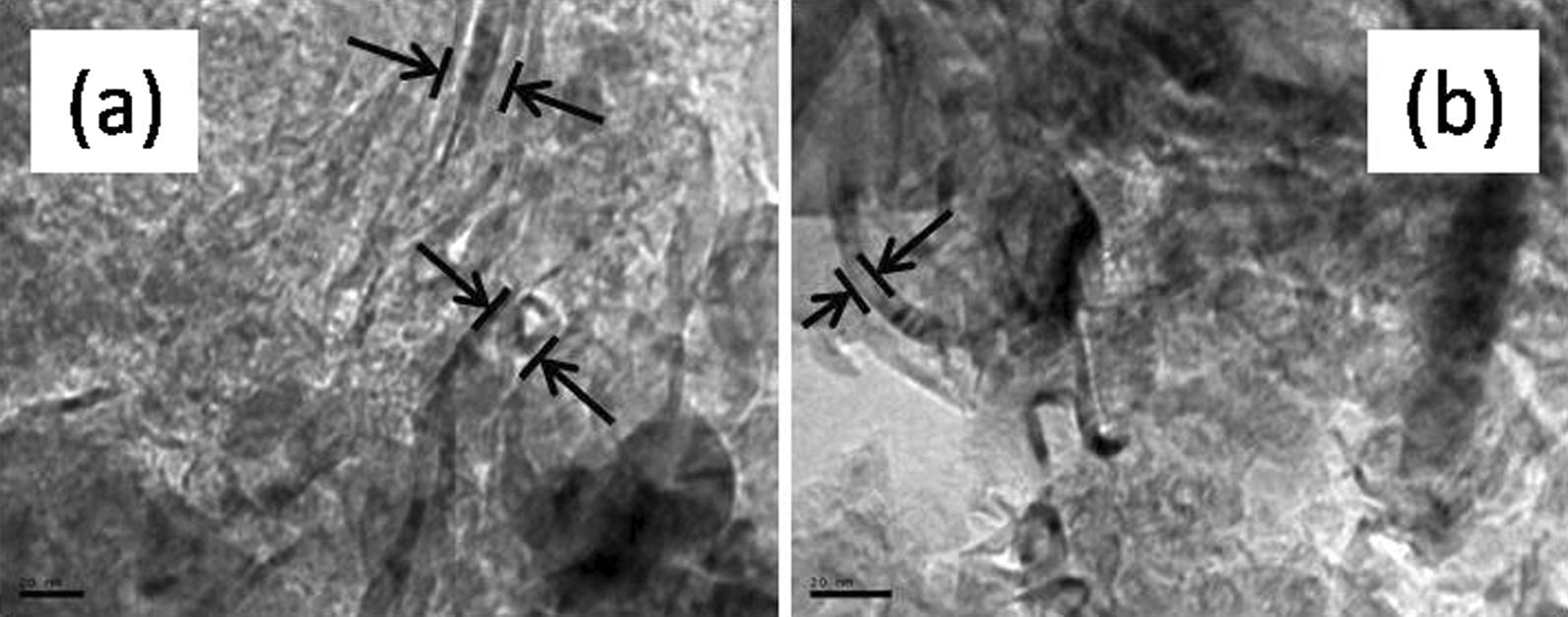
TEM images of studied CNF samples. Arrows indicate the edges of the fibers

The carbon nanofibers treated at 860 °C do not undergo any significant change and keep their original fiber structure. Figure 1 shows two images of studied fibers after the treatment at this temperature in vacuum. Some individual fibers can be clearly observed. The diameter of the observed fibers is clearly below 50 nm, but this is just an effect arising from the magnification used: ×400 k for Fig. 1a and ×500 k for Fig. 1b. The larger fibers are often tangled with each other, and they are not easy to be discriminated at these magnifications. In other works, in which the processing temperature was significantly higher, the presence of the individual nanofibers was shown at lower magnifications [34]. The reason to follow this treatment is to allow a correct and easy handling of the nanofibers in the different experiments performed without modifying their structure. The conductivity of the SPSed fibers is 10^2^ (Ω cm)^−1^ as stated in previous works. [34].

### X-Ray Photoelectron Spectroscopy

**Fig. 2:**
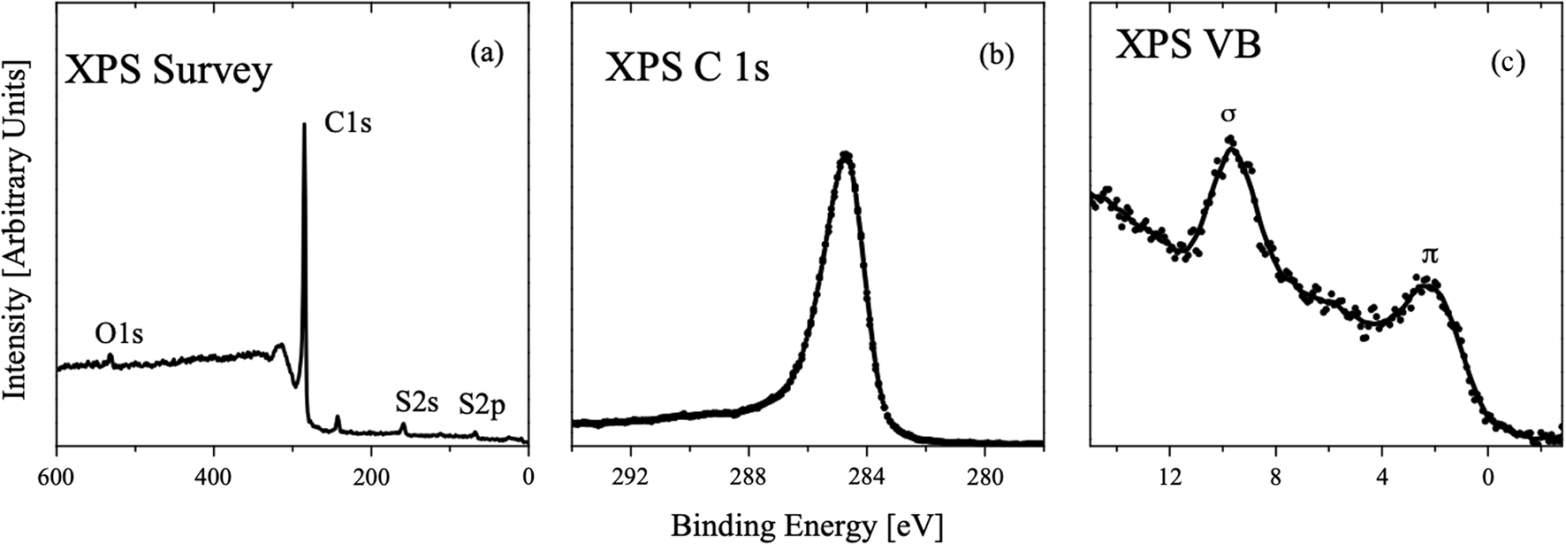
XPS survey (**a**), C 1*s* **b** valence band and **c** spectra of CNF

The surface composition of CNF determined from the XPS survey spectrum (see Fig. 2a and Table 1) demonstrates the presence only small impurities of oxygen, nickel and sulfur impurities. The high-energy resolved XPS C 1* s* (Fig. 2b) has a binding energy of 284.7 eV, which is typical for sp^2^ carbon of graphene [35] and confirms that the main characteristic of nanofibers is the stacking of graphene sheets of varying shape [36], which is in agreement with TEM picture (Fig. 1). It exhibits the high-energy asymmetric tailing and a weak plasmon satellite 6–7 eV apart from the parent C l*s*-line also showing the presence of carbon atoms with sp^2^-like bonding symmetry [37]. The presence of Ni and S is due to use of Ni catalyst and H_2_S-gas during the synthesis of the carbon nanofibers. The O/C ratio determined from XPS survey spectrum is 0.019, which explains the absence of any C–O functional groups and allows attributing CNFs to highly hydrophobic materials. On the one hand, it restricts some adsorption applications and even the special methods are developed for their chemical activation [38] and, on the other hand, the hydrophobic property is very attractive and suitable for special applications of CNFs such as conducting fillers of ceramic materials.

**Table 1:** Surface composition (in at.%)

Sample	C	O	Ni	S
Carbon nanofiber	94.8	1.8	1.6	1.8

The existing studies of chemical bonding and electronic structure of CNFs are mostly restricted by photoemission measurements and DFT calculations of oxidized materials [38–41]. The XPS valence band of CNF (Fig. 2c) consists of two main *π*- and *σ*-peaks located at 2.3 and 9.6 eV, respectively. We will discuss later the origin of these peaks based on our DFT calculations. For the moment, we will just mention that the intensity distribution near the Fermi level certainly indicates that CNF is a conducting material.

### Raman Measurements

**Fig. 3:**
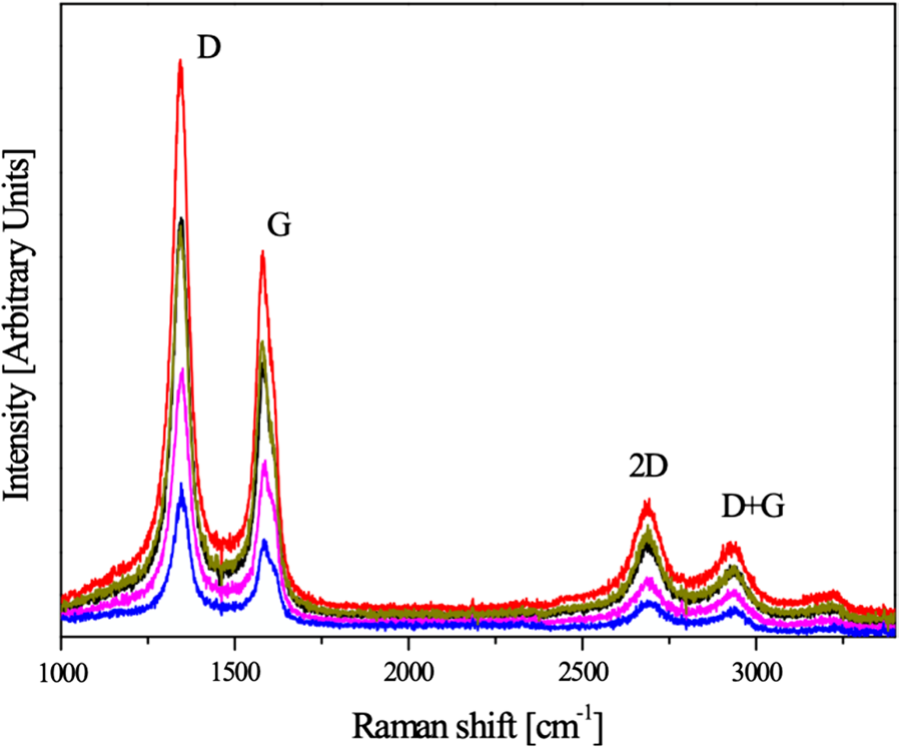
Raman spectra taken in 5 different places of carbon fiber sample

Measured Raman spectra (Fig. 3) are found to be different from that of single-layer graphene, graphite, carbon nanotubes [42] or graphene oxide [43, 44]. The presence of *D* + *G* and *D* peaks evidence near planar structure of studied materials in contrast to CNT where these peaks are not observed. The absence of *G* peak in Raman spectra of graphite and the presence of distinct *G* peak in spectra of all studied samples demonstrate the absence of graphitization. In contrast to Raman spectra of graphene oxide where 2D and *D* + *G* peaks are broad and sometimes undistinguished, the same peaks in Fig. 3 can be described as rather narrow. The key difference with Raman spectra of graphene is the presence of *D* + *G* peak and absence of high and sharp 2D peak. The combination of distinct *D*, *G* peaks with traces of *D*′ peak and less distinct 2D and *D* + *G* peaks makes these spectra similar to those observed in nanocrystalline carbon [43]. Significant magnitude of *D* peak corresponds to distortions of graphenic sheets similarly to observed for wrinkled graphene sheets exfoliated by different methods [45]. Thus, based on Raman spectra we can exclude oxidation of CNF even as formation of ordered layered structures such as graphene multilayers.

### Theoretical Modeling

**Fig. 4:**
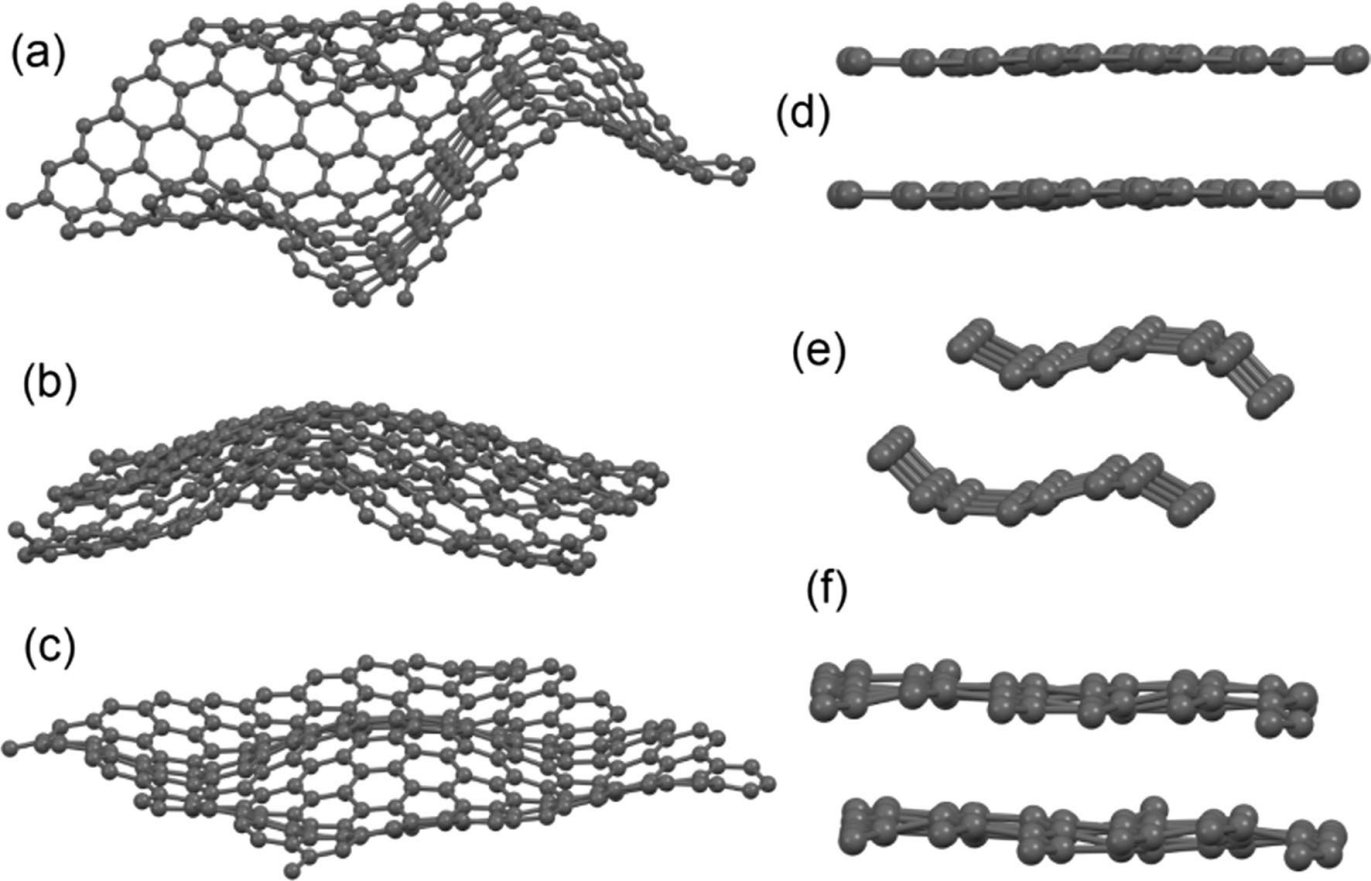
Optimized atomic structures of different graphene monolayers (**a**–**c**) and bilayers (**d**–**f**) with distortions of different shapes and sizes

To unveil the atomic structure of the stacked-cup CNF, we performed the calculations of various carbon nanostructures. Based on the results discussed above of TEM, Raman and XPS measurements, we exclude disordered kinds 3D carbons and carbon nanotubes and considered only flat and distorted graphene mono- and bilayers. To create the distortions of various shapes and sizes, we compressed in-plane graphene membrane and shifted some atoms in the central part of the plane. The further relaxation provides the restoring carbon–carbon distances by the formation of visible out-of-plane distortions (see Fig. 4). The size and shape of distortion depend on the magnitude of the initial compression. To imitate the bending (Fig. 4e), the initial compression was along one of the axis; in other cases, the initial compression was uniaxial.

**Fig. 5:**
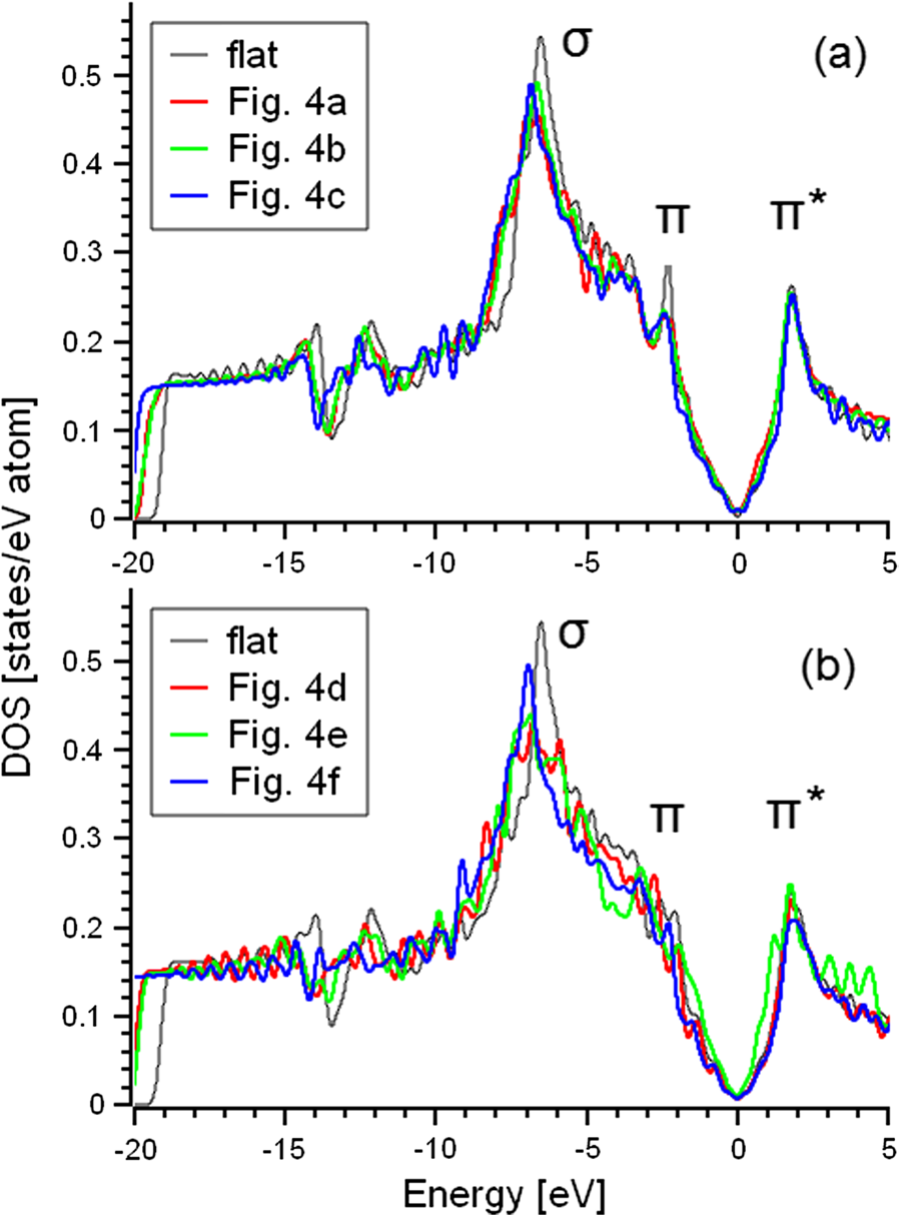
Density of states for flat and distorted monolayers (**a**) and bilayers (**b**) shown in Fig. 3. Fermi level set as zero

The results of the calculation demonstrate that the distortion of both mono- and bilayers provides shift down of σ-peak at about 1 eV (see Fig. 5). The origin of this shift is the increasing localization of the electrons on these orbitals caused by distortions-induced changes in the crystal field. There is no visible difference between mono- and bilayers because these orbitals are related to in-plane covalent bonds. This result is in rather good agreement with experimental XPS valence band spectra (Fig. 2c). Since *π*-orbitals are oriented out of plane and create a *π*–*π* interlayer bond, the influence of the distortions on *π*-peak is quite significant only for bilayers (Fig. 4b). Even tiny distortions of the bilayer (such as shown in Fig. 4d) provide the broadening of the *π*-peak and merging of this distinct feature of the electronic structure the with upper edge of the *σ*-peak. The origin of these changes in the electronic structure is due to the formation of multiple *π*–*π* interlayer bonds in areas with different distortions. We can conclude that the studied CNFs are mainly composed from various distorted graphene monolayers and the contribution from layered structures is insignificant because the experimental spectra (Fig. 2c) present a distinctive *π*-peak. Because the distortion of the graphenic sheets influences its catalytic properties [28], it should be taken into account in the theoretical modeling of catalytic activity of CNF [12, 13].

### Optical Properties of CNFs

**Fig. 6:**
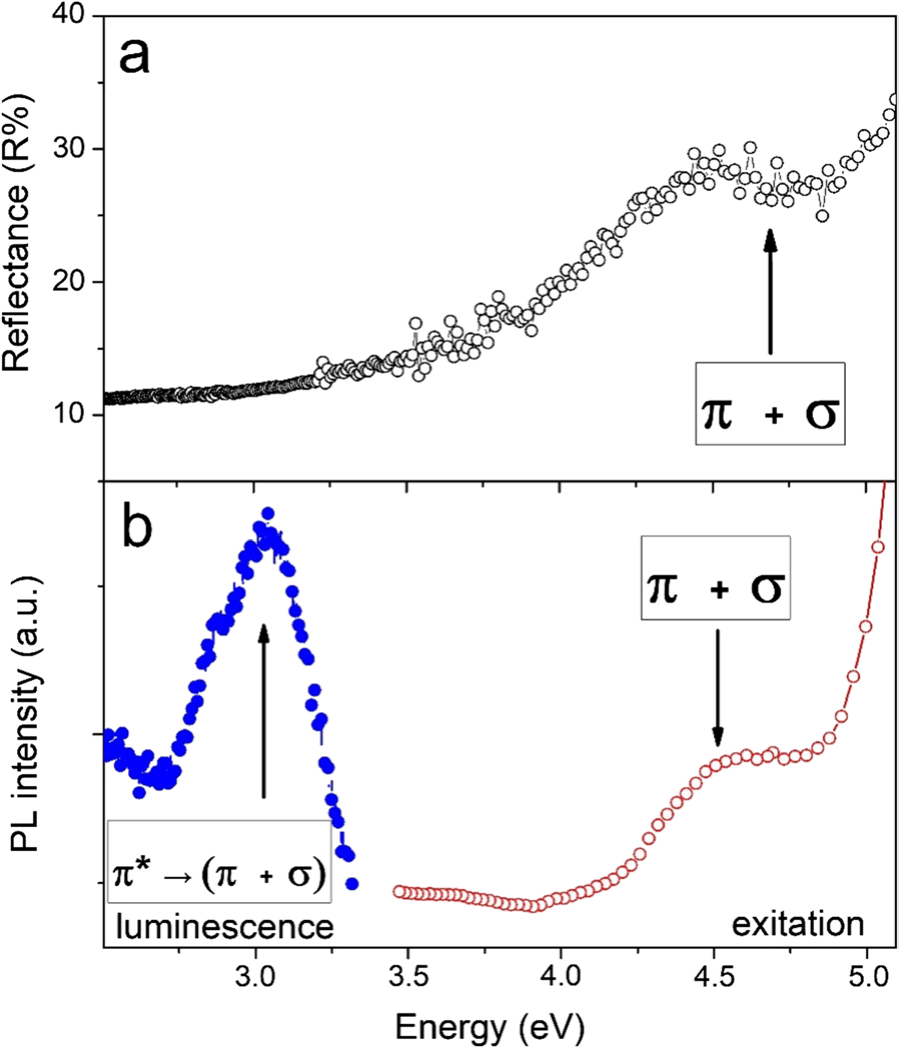
Reflectance (**a**) and photoluminescence (**b**) spectra of CNF. The arrows indicate the contribution of ground **π** and exited π* states of GQDs in excitation–relaxation process

Additional measurements of the reflectance spectra of the studied samples (Fig. 6a) demonstrate weak reflections in the visible and IR ranges and a weak adsorption in UV region at 260 nm (4.75 eV). The observed weak reflection can be interpreted as combination of two factors. The first is deviation of conductance from Drude model and therefore unsuitability of Hagen–Rubens relation for description of the reflectance of these compounds [46]. The second is macroscopic disorder of graphenic sheets that provides light trapping by multiple reflections as it was discussed for CNT forests [47, 48]. The presence of the peculiarity in the UV part of spectra is related to the presence in the composite of some amount of materials with an energy gap. Such a material with an energy gap can most likely be represented by a low-dimensional carbon phase. Amongst the possible candidates, in our case, the most suitable are graphene quantum dots, whose characteristic property is the presence of photoluminescence [24]. In this regard, we additionally performed measurements of the photoluminescence characteristics of the samples under study. Measurements of the photoluminescence spectra (Fig. 6b) reveal the presence of the peak at 420 nm (3 eV). A narrow UV band with a maximum at 270 nm (4.5 eV) was found in the spectra of excitation (PLE) related to this peak. Because similar values of optical transitions were obtained for GQDs [24, 49], we can exclude oxide contaminants as a source of the optical activity of CNFs. In contrast to chemically synthesized GQDs [29], the selective *σ* – π* transition at 4.36 eV is absent in the studied CNFs samples. The possible explanation of this fact is the broadening of the band corresponding with *π* – π* transition and the intensive adsorption in the spectra of diffusive reflectance at 240 nm caused by the overlap between σ and *π* bands.

**Fig. 7:**
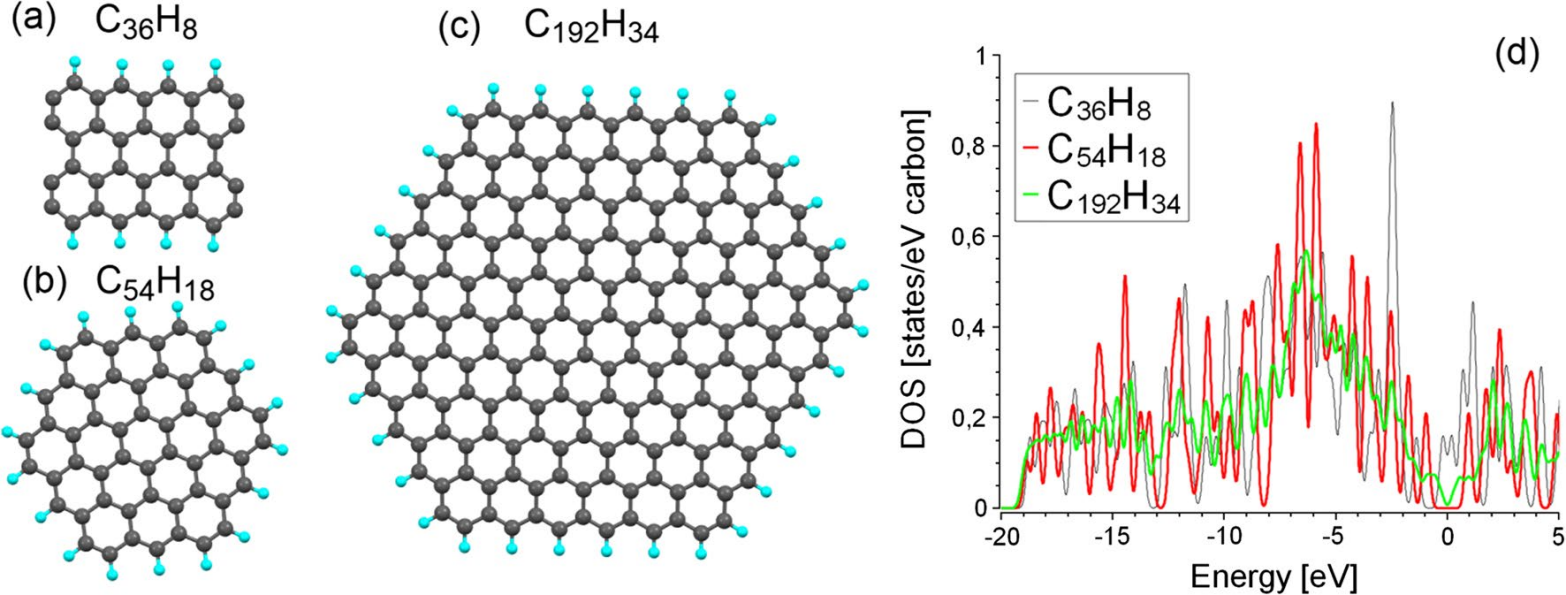
Optimized atomic structure (**a**–**c**) and total densities of states (**d**) for selected nanographenes. Carbon and hydrogen atoms on panels **a**–**c** are shown in dark grey and cyan colors, respectively. Fermi level on panel d set as zero

In order to explain the combination of the presence of the contribution from GQDs in optical spectra and graphene like valence bands and Raman spectra (see 2.1–2.3), we have performed an additional set of theoretical modeling. Because XPS spectra (Fig. 2b) evidence the absence of oxidation and *sp*3 hybridization, we used for this purpose only flat nanographenes with edges passivated by hydrogen atoms (Fig. 7a–c). Note that all carbon atoms in these nanographenes are in *sp*2 hybridization. For all these systems, we performed optimization of the atomic positions with further calculations of the electronic structure (Fig. 7d). The absence of dangling bonds on the edges was checked by including of spin-polarization and Mulliken population analysis. Calculated deviations of carbon–carbon distances from the values in distorted graphene sheets are less than 0.01 Å. Results of the calculations evidence the presence of the bandgap in nanographenes of sizes above 1 nm (C_54_H_18_ and C_192_H_34_). The difference of the shape and size of nanographenes influence only the value of the bandgap. The electronic structure of the valence bands and position of the π* peak in conductive bands is similar to distorted graphene (see Fig. 5a). This result explains the absence of a visible contribution from GQDs in VB spectra (Fig. 2b). Note that the electronic structure of GQDs demonstrates a larger overlap between σ and *π* bands that is in qualitative agreement with the results of optical measurements.

## Conclusions

Complementary DFT, XPS and optical spectra studies have shown that stacked-cup CNFs are mainly composed of various distorted graphene monolayers. The contribution in electronic structure from layered structures and some amount of nanographenes is insignificant. Delocalized *π*-electrons are oriented out of plane and create *π*–*π* interlayer bonds, and the influence of the distortions on *π*-peak is quite significant only for bilayers. These delocalized *π*-electrons can freely move throughout the structure providing a good electrical conductivity, which is of great importance for multiple applications from electronics to composites. The presence of nanographenes leads to the appearance of the optical transitions in UV spectra. The combination of outstanding electrical properties and optical transitions makes the CNFs promising materials with possible prospects for controlling the electronic properties of composites ranging from conductors to materials with an energy gap.

## Data Availability

All raw data from measurements, input and output data for calculations and used samples are available by request to authors.

## Abbreviations

CNFs: Carbon nanofibers
DFT: Density functional theory
GQD: Graphene quantum dots
IR: Infrared
QD: Quantum dots
UV: Ultraviolet
XPS: X-ray photoelectron spectroscopy
XRD: X-ray diffraction
